# Realistic simulation: construction and validation of scenarios for stroke recognition in prehospital care

**DOI:** 10.1055/s-0046-1817027

**Published:** 2026-03-19

**Authors:** Bruna Caroline Gorla, Meire Cristina Novelli e Castro, Sarah Vendramini Vidotto, Gabrielle Pires de Campos, Priscila Masquetto Vieira de Almeida

**Affiliations:** 1 Universidade Estadual Paulista “Júlio de Mesquita Filho,” Faculdade de Medicina de Botucatu, Departamento de Enfermagem, Botucatu SP, Brazil.

**Keywords:** Prehospital Care, Stroke, Simulation Training, Education, Continuing, Validation Study

## Abstract

**Background:**

Stroke remains one of the leading causes of morbidity and mortality worldwide, requiring rapid and accurate recognition, especially in the context of prehospital care.

**Objective:**

To construct, validate, and produce simulated scenarios for teaching and learning about the care of stroke victims in prehospital settings.

**Methods:**

The present is a methodological, descriptive, and quantitative study involving the development of two simulated scenario scripts based on best simulation practices and Bloom's taxonomy. Validation was conducted by 11 expert judges selected according to Fehring's criteria, using an adapted instrument and the Content Validity Index (CVI), with a value of 0.90 or higher considered valid.

**Results:**

All evaluated items of the scripts achieved full agreement among the judges, with a mean CVI of 1.0. The scenarios incorporate both typical and atypical signs and symptoms of stroke and are applicable for training prehospital care teams.

**Conclusion:**

The validated simulated scenarios have the potential to enhance the training of prehospital care professionals, promoting the early recognition of stroke across different regional contexts in Brazil.

## INTRODUCTION

According to the Global Burden of Diseases, 1 stroke remains the second leading cause of death and the third leading cause of morbidity and mortality worldwide, with higher prevalence in low- and middle-income countries. In Brazil, the age-adjusted prevalence rate reached 1,133 cases per 100 thousand inhabitants in 2017. 2

Stroke is a disabling disease with high morbidity and mortality, characterized by a sudden-onset neurological deficit caused by an interruption of cerebral blood flow due to either ischemia or hemorrhage. Thus, strokes are classified as ischemic or hemorrhagic, with the former being more prevalent. Initial treatment for ischemic stroke is time-dependent and should be initiated within 4.5 hours of symptom onset. Delayed initiation of therapy is associated with higher mortality rates and poorer clinical outcomes. 3 4 5

In Brazil, most of the cases attended by the Urgent Mobile Care Service (Serviço de Atendimento Móvel de Urgência, SAMU, in Portuguese) involve clinical emergencies, including the care of suspected stroke victims. Prehospital care is essential in the acute phase of the disease, as these teams are qualified to perform early recognition and appropriate stabilization of the patient, as well as refer them for diagnosis and treatment. However, accurately identifying the signs and symptoms of stroke remains challenging, as they may resemble those of other cardiovascular conditions. 6 7 8

In this context, it is important to provide training for professionals working in this area. Various methodologies can be used; however, realistic simulation has gained prominence in recent years due to its potential to train clinical skills practically, allowing participants to experience real-life scenarios without putting patients or learners at risk. Realistic simulation scenarios enable the practice of decision-making, leadership, teamwork improvement, and communication. 9 10 11 Nevertheless, national studies exploring the development and validation of realistic simulation scenarios for stroke recognition in this context are still scarce.

Therefore, the current study aims to construct, validate, and produce two simulated scenarios for teaching and learning about the care of stroke victims in prehospital settings.

## METHODS

### Study design

The present is a methodological, descriptive study with a quantitative approach, aimed at constructing and validating two simulated scenarios. The study was conducted at SAMU, located in the city of Botucatu, in the state of São Paulo, Brazil. The primary outcome was the validation of the scenario scripts, assessed using the Content Validity Index (CVI), a statistical measure that evaluates the degree of agreement among experts regarding the relevance and representativeness of each item. 12

The study was conducted in two phases. The 1st involved the construction and validation of 2 simulated scenarios by expert judges in the field (from September 2023–January 2024), and the 2nd consisted of the production of the simulated scenarios with the SAMU team (from February– March 2024).

The construction of the simulated scenarios was based on the best practice guidelines for simulation design recommended by the International Nursing Association for Clinical Simulation and Learning, 13 the scenario construction framework by Fabri et al., 14 and Bloom's taxonomy, 15 which relates educational objectives to cognitive, affective, and psychomotor domains.

### Participants and inclusion and exclusion criteria

The validation of the simulated scenario scripts was conducted by expert judges in the field, including nurse professionals and faculty members with specialist training, a master's and/or doctoral degree, clinical experience in the subject, and related scientific publications. The inclusion criteria for classifying a participant as an expert judge were adapted from Fehring's method, 16 requiring a minimum score of 5 points to be classified as an expert.

The validation of the scenario scripts was performed by 11 expert judges in the field, all of whom were nurses, 10 female and 1 male, with a mean age of 45.5 years. Regarding academic degrees, 2 (18.2%) held a master's degree, and 9 (81.8%) held a doctoral degree. In terms of professional activity, 3 (27.3%) worked exclusively in university teaching, 2 (18.2%) exclusively in healthcare institutions, 4 (36.3%) in both areas, and 2 (18.2%) in other fields. The mean professional experience among participants was 19.6 years.

Regarding experience and/or management of prehospital care services, 5 judges (45.5%) reported such experience, with a mean duration of 11.2 years. All judges (100%) reported experience in caring for patients affected by stroke, and 8 (72.7%) had experience in the development and evaluation of simulated scenarios.

The selection of expert judges was conducted by convenience sampling, via a search on Plataforma Lattes of Conselho Nacional de Desenvolvimento Científico e Tecnológico (CNPq), and they were external professionals to the research. The number of judges followed literature recommendations for content validation, with a minimum of 5 expert judges. 17

To minimize potential biases, strict and objective criteria were adopted for the selection of expert judges. Additionally, each judge independently evaluated the scenario scripts, that is, without prior knowledge of the results obtained by the other judges, using a previously validated instrument. Suggestions and all discrepancies among expert judges were analyzed and incorporated into the final product following discussion and consensus.

The participants in the simulated scenarios included ambulance drivers, nursing technicians, and nurses working in day and night shifts. Inclusion criteria were being over 18 years of age, employed by SAMU, and voluntarily agreeing to participate in all phases of the study. Interns and professionals who did not participate in the realistic simulation due to being on vacation or leave of absence were excluded.

### Data collection

For the validation of the simulated scenarios, data collection was conducted using an adapted instrument for the expert judges in the field. 18 19

The expert judges evaluated the scenario scripts by indicating their level of agreement with each item in the instrument, using a Likert scale ranging from 1 to 4: “disagree,” “partially disagree,” “partially agree,” and “agree.” Each item included a space for comments and justifications, if the response was “disagree,” “partially disagree,” or “partially agree,” as well as for any additional descriptive contributions.

We invited each expert judge to participate in the validation process of the simulated scenarios by email, clearly explaining the objective of the study. Those who agreed to participate completed the informed consent form, the biographical and professional characterization questionnaire, and the adapted instrument, all provided through Google Forms.

The SAMU team members who agreed to participate in the simulation were asked to complete the Informed Consent Form, a biographical and professional characterization questionnaire, and the simulation design scale, all in an online format.

### Data analysis

For the validation of the simulated scenarios, the CVI was calculated to assess agreement regarding the representativeness of each item. The CVI corresponds to the proportion (%) of judges who responded “agree” or “partially agree” relative to the total number of judges. In this study, a minimum acceptable index of 0.90 was established for each item. 12 Descriptive analysis was performed through a careful review of the judges' comments and suggestions, and the scenario scripts were modified when appropriate, using Microsoft Excel (Microsoft Corp.). All data were stored on password-protected institutional computers with restricted access to the research team.

**Figure 1:**
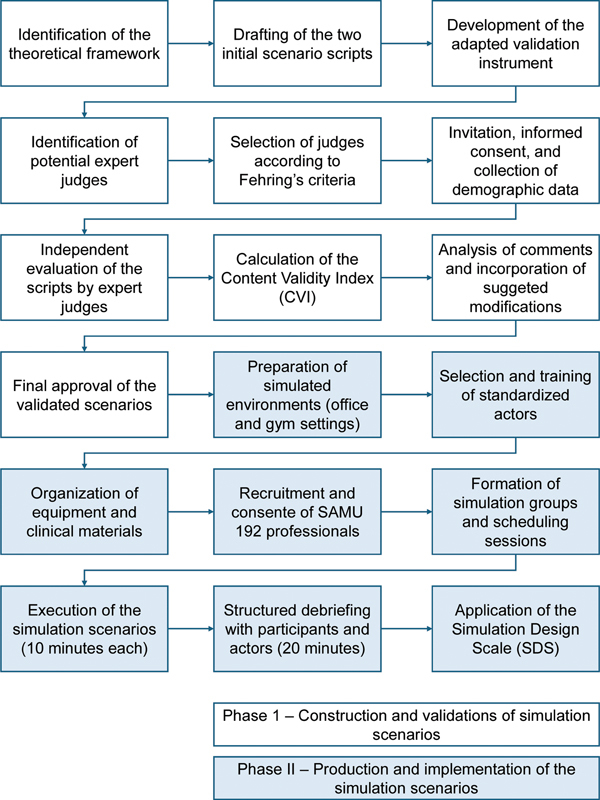
Flow diagram of the study phases.

Figure 1 shows the flow diagram of the study, detailing the two phases of construction, validation, and production of the simulated scenarios.

### Ethical and legal considerations

Ethics approval was granted by Faculdade de Medicina de Botucatu's Research Ethics Committee (Protocol: 6,292,428).

No artificial intelligence tools were used in any stage of data collection, data analysis, scenario construction, or interpretation of the results. Artificial intelligence tools (ChatGPT, OpenAI) were used exclusively for English language editing, grammar refinement, and improvements in textual clarity. No AI system contributed to the scientific content, methodological decisions, or conclusions of the study.

## RESULTS

Two scripts were developed for the construction of the simulated scenarios, one for each scenario. The first script was titled “Prehospital care for stroke victims: recognition of typical signs and symptoms,” and the second, “Prehospital care for stroke victims: recognition of atypical signs and symptoms.” Both scripts consisted of 19 items and are available as **Supplementary Material Tables S1** and **S2** (available at https://www.arquivosdeneuropsiquiatria.org/wp-content/uploads/2025/12/ANP-2025.0271-Supplementary-Material.docx ), including all modifications made by the expert judges after the validation process.

Accordingly, the scripts included items addressing participants' prior knowledge, general and specific objectives, theoretical foundation, scenario topic, authorship, scenario complexity, simulation modality, scenario duration, required documents, briefing, clinical case description, human and material resources used in the scenario, characterization of actors, target audience, team training for the activity, debriefing, and scenario evaluation.

The adapted instrument used by the judges for content validation of the simulated scenarios consisted of 14 items. Tables 1 and 2 present the items of the instrument and the respective CVI calculations for each scenario.

**Table 1:** Evaluation items for the first scenario “Prehospital Care for Stroke Victims: Recognition of Typical Signs and Symptoms”

Item	Completely inadequate		Inadequate but can be revised		Adequate with minor adjustments		Completely adequate		CVI
	n	%	n	%	n	%	n	%	
Clinical case plausibility	–	–	–	–	2	18.2	9	81.8	1.0
Realism	–	–	–	–	–	–	11	100	1.0
Adherence to available scientific evidence	–	–	–	–	1	9.1	10	90.9	1.0
Complexity relative to the knowledge and skills level of professionals	–	–	–	–	–	–	11	100	1.0
Case description	–	–	–	–	1	9.1	10	90.9	1.0
Information provided to professionals before the simulation	–	–	–	–	1	9.1	10	90.9	1.0
Learning objectives	–	–	–	–	–	–	11	100	1.0
Promotion of autonomous problem solving	–	–	–	–	2	18.2	9	81.9	1.0
Promotion of critical thinking	–	–	–	–	2	18.2	9	81.9	1.0
Simulated environment	–	–	–	–	2	18.2	9	81.9	1.0
The actor parameters are consistent with the clinical case	–	–	–	–	2	18.2	9	81.9	1.0
Materials and equipment available to professionals	–	–	–	–	1	9.1	10	90.9	1.0
Checklist of expected actions	–	–	–	–	3	27.3	8	72.7	1.0
Aspects evaluated in the debriefing	–	–	–	–	–	–	11	100	1.0
**Mean CVI**	**1.0**								

Abbreviation: CVI, Content Validity Index.

**Table 2:** Evaluation items for the second scenario “Prehospital Care for Stroke Victims: Recognition of Atypical Signs and Symptoms”

Item	Completely inadequate		Inadequate but can be revised		Adequate with minor adjustments		Completely adequate		CVI
	n	%	n	%	n	%	n	%	
Clinical case plausibility	–	–	–	–	1	9.1	10	90.9	1.0
Realism	–	–	–	–	1	9.1	10	90.9	1.0
Adherence to available scientific evidence	–	–	–	–	1	9.1	10	90.9	1.0
Complexity relative to the knowledge and skills level of professionals	–	–	–	–	1	9.1	10	90.9	1.0
Case description	–	–	–	–	2	18.2	9	81.8	1.0
Information provided to professionals before the simulation	–	–	–	–	2	18.2	9	81.8	1.0
Learning objectives	–	–	–	–	–	–	11	100	1.0
Promotion of autonomous problem solving	–	–	–	–	2	18.2	9	81.8	1.0
Promotion of critical thinking	–	–	–	–	2	18.2	9	81.8	1.0
Simulated environment	–	–	–	–	–	–	11	100	1.0
The actor parameters are consistent with the clinical case	–	–	–	–	2	18.2	9	81.8	1.0
Materials and equipment available to professionals	–	–	–	–	1	9.1	10	90.9	1.0
Checklist of expected actions	–	–	–	–	3	27.3	8	72.7	1.0
Aspects evaluated in the debriefing	–	–	–	–	–	–	11	100	1.0
**Mean CVI**	**1.0**								

**Abbreviation:** CVI, Content Validity Index.

All items in both simulated scenarios achieved CVI values above the minimum recommended threshold of 0.90, with each item and the overall CVI reaching 1.0. At the end of each item, judges were able to provide comments, questions, and suggestions. All comments were analyzed and incorporated when relevant.

For both scenarios, the judges suggested reviewing the role of nursing technicians within the scenarios, selecting actors with health backgrounds for better understanding of the cases, and making minor spelling corrections in the clinical narrative. For the atypical scenario, one judge suggested adjusting the patient's profession and adding oxygen saturation information. All modifications were accepted and integrated into the scripts.

Table 1 shows the CVI results for the first scenario (typical signs and symptoms) and Table 2 presents the results for the second scenario (atypical signs and symptoms).

The simulated scenarios were produced in settings that reflect the environments encountered daily by prehospital care professionals. To represent the first scenario, an office environment was set up using a desk, chair, rug, ottoman, bookshelf, and office supplies such as folders, a lamp, notebooks, pens, among others. The second scenario depicted a gym, equipped with a mat, exercise ball, dumbbells, resistance bands, ankle weights, and other items. All materials and equipment necessary for victim care were made available to the participants. Additionally, two standardized actors participated in both scenarios: one representing the patient and the other the companion.

A total of 25 professionals from both day and night shifts participated in the simulation activity, including 11 ambulance drivers, 9 nursing technicians, and 5 nurses. Of these, 14 were male and 11 were female, with a mean age of 39 years. The average duration of professional experience in healthcare was 9.3 years. Nearly all participants, except for 1, reported providing care to stroke victims, with 36% indicating a high frequency of such cases. When asked whether they feel insecure when providing care to stroke victims, more than half (60%) responded affirmatively.

The simulation activity took place over 2 separate days, with alternative time slots offered each day to accommodate the largest possible number of participants. As a result, four groups were formed. The activity was structured so that participants were gathered in a room with two facilitators. In each group, two professionals participated directly in the scenarios, one pair for each scenario, on a voluntary basis, while the remaining participants observed the scenes from another room via a large screen. The selected pair was guided by a facilitator who remained inside the scenario setting. Each scenario was conducted within a set time frame of 10 minutes.

After the completion of the simulation activity, all participants, including the actors, were brought together for a discussion of each scenario. A structured debriefing was conducted, during which all individuals involved in the activity raised questions, highlighted positive aspects and areas for improvement, and provided feedback, both from participants and actors. This stage lasted 20 minutes.

Finally, for the evaluation of the simulation, 18 professionals—representing 72% of the participants—completed the Simulation Design Scale, 20 which covers the following themes: objectives and information, support, problem-solving, feedback/reflection, and realism, for a total of 20 items. Most items received full agreement; only two participants indicated difficulty with the level of the simulation and one with the scenario itself but noted that their concern was addressed and clarified.

## DISCUSSION

In the present study, two realistic simulation scenarios focused on the recognition of typical and atypical signs and symptoms of stroke in prehospital care were developed, validated, and produced. Validation was conducted by 11 expert judges, following strict selection criteria, resulting in full agreement on all evaluated items (mean CVI of 1.0). These results demonstrate strong methodological rigor and highlight the scenarios' practical applicability for training SAMU teams across Brazil, with the potential to enhance the quality of prehospital stroke care.

Despite advances in medicine, stroke remains one of the leading causes of death and morbidity worldwide, and early recognition is essential for rapid initiation of treatment. Simulation-based training has proven effective in improving knowledge and clinical competencies, strengthening decision-making, and increasing professionals' confidence in emergency situations, ultimately contributing to improved patient outcomes. 21

Regarding the selection of judges, the literature emphasizes the importance of clinical expertise and scientific contribution. 17 In the current study, all judges had experience in stroke care, and most also had academic experience, which reinforced the quality and consistency of the validation process. The CVI is a widely used measure for assessing agreement among experts, and values above 0.90 are recommended for validating educational tools. 12 17 In this study, both scenarios achieved a CVI of 1.0, reflecting unanimous agreement on the relevance and adequacy of the items.

The development of the scenarios followed best-practice guidelines for simulation design, including clear learning objectives, realistic case construction, defined participant roles, structured debriefing, and post-simulation evaluation. 13 22 Bloom's taxonomy was used as a guiding framework to ensure alignment between the learning objectives and the cognitive, affective, and psychomotor dimensions required for clinical practice. 15 19 23

The debriefing phase, conducted immediately after the scenarios, was essential to support reflective learning and consolidate key competencies. A structured debriefing allows participants to analyze their decisions, identify knowledge gaps, and improve technical and non-technical skills within a safe environment. 13 24

The Simulation Design Scale results demonstrated high participant satisfaction and confirmed that the scenarios provided opportunities to practice critical thinking, decision-making, and technical skills, elements considered essential for a well-designed simulation activity. 25 26

The validated scenarios demonstrated broad applicability and can be implemented in all SAMU units throughout Brazil. Designed to train the recognition of stroke signs and symptoms, the scenarios are suitable for both Basic Life Support (BLS) and Advanced Life Support (ALS) teams. This is particularly important given that BLS teams represent ∼ 83% of SAMU mobile units in the country. 27 Ensuring that BLS professionals are well trained to identify stroke is crucial for reducing delays in care and improving clinical outcomes, especially in regions with limited ALS coverage.

The current study contributes to addressing the existing gap in validated, specific educational tools for prehospital stroke care in Brazil. By demonstrating that it is feasible to construct and validate realistic simulation scenarios with high expert agreement, this research strengthens the basis for implementing systematic training programs within SAMU.

These findings align with international evidence supporting simulation as an effective strategy for developing clinical competencies in time-sensitive emergencies. Incorporating validated scenarios into continuing education efforts may reduce errors in stroke recognition and improve patient triage and referral, particularly within BLS teams, ultimately benefiting the stroke care pathway nationwide.

The main limitation of the present study lies in the absence of an evaluation phase assessing the practical impact of the validated scenarios on learning retention and clinical performance. Although the scenarios were developed and validated with methodological rigor, their effectiveness in real-world practice was not measured. Future studies should investigate the longitudinal impact of these scenarios on professional performance and patient outcomes in stroke care.

In conclusion, the current study successfully achieved its objective of constructing, validating, and producing simulated scenarios for the recognition of typical and atypical signs and symptoms of stroke, with validation resulting in the highest CVI and full agreement among expert judges. The involvement of judges with expertise in the field was essential to ensure the theoretical and practical relevance of the scenarios. It is expected that these scripts will be widely used as a continuing education resource for prehospital care professionals across different regions of the country, contributing to faster, more accurate, and higher-quality care for stroke patients. Future research is recommended to evaluate the impact of these scenarios on clinical practice and stroke care outcomes.
